# A brain-inspired robot pain model based on a spiking neural network

**DOI:** 10.3389/fnbot.2022.1025338

**Published:** 2022-12-20

**Authors:** Hui Feng, Yi Zeng

**Affiliations:** ^1^Brain-inspired Cognitive Intelligence Lab, Institute of Automation, Chinese Academy of Sciences, Beijing, China; ^2^School of Artificial Intelligence, University of Chinese Academy of Sciences, Beijing, China; ^3^Center for Excellence in Brain Science and Intelligence Technology, Chinese Academy of Sciences, Shanghai, China; ^4^National Laboratory of Pattern Recognition, Institute of Automation, Chinese Academy of Sciences, Beijing, China

**Keywords:** brain-inspired intelligent robot, robot pain, spiking neural network, free energy principle, spike-time-dependent-plasticity

## Abstract

**Introduction:**

Pain is a crucial function for organisms. Building a “Robot Pain” model inspired by organisms' pain could help the robot learn self-preservation and extend longevity. Most previous studies about robots and pain focus on robots interacting with people by recognizing their pain expressions or scenes, or avoiding obstacles by recognizing dangerous objects. Robots do not have human-like pain capacity and cannot adaptively respond to danger. Inspired by the evolutionary mechanisms of pain emergence and the Free Energy Principle (FEP) in the brain, we summarize the neural mechanisms of pain and construct a Brain-inspired Robot Pain Spiking Neural Network (BRP-SNN) with spike-time-dependent-plasticity (STDP) learning rule and population coding method.

**Methods:**

The proposed model can quantify machine injury by detecting the coupling relationship between multi-modality sensory information and generating “robot pain” as an internal state.

**Results:**

We provide a comparative analysis with the results of neuroscience experiments, showing that our model has biological interpretability. We also successfully tested our model on two tasks with real robots—the alerting actual injury task and the preventing potential injury task.

**Discussion:**

Our work has two major contributions: (1) It has positive implications for the integration of pain concepts into robotics in the intelligent robotics field. (2) Our summary of pain's neural mechanisms and the implemented computational simulations provide a new perspective to explore the nature of pain, which has significant value for future pain research in the cognitive neuroscience field.

## 1. Introduction

Pain is vital to individual organisms' survival and the social life between organisms (Walters and Williams, 2019). The International Association for the Study of Pain (IASP) defines pain as “an unpleasant sensory and emotional experience associated with, or resembling that associated with, actual or potential tissue damage” (Raja et al., 2020). Pain can alert to actual injuries and prevent potential injuries, help organisms protect themselves (Hardcastle, 1997; Loeser and Melzack, 1999), and synergistically trigger various cognitive functions, such as pain memory and pain empathy (Jackson et al., 2006; Wiech and Tracey, 2013; Asada, 2019). Designing Robot Pain inspired by the organisms' pain has essential implications for the survival and longevity of robots.

Previous works related to robots and pain have focused on how robots recognize pain signals, such as humans' painful expressions, and realize pain empathy for humans (Cao et al., 2021; Werner et al., 2022). Kuehn and Haddadin (2016) designed the robot's neural reflex behavior for pain, which is only a pain-induced avoidance response and not a human-like pain capacity. Sur and Amor (2017) attempted to model the robot's pain capacity, but the network structure they used is different from the biological mechanism. We explored the neural mechanisms of pain and established a Brain-inspired Robot Pain Spiking Neural Network (BRP-SNN) to simulate the brain regions involving pain, using the spike-timing-dependent-plasticity (STDP) learning rule to train the connection weights. Our model makes the robots have human-like pain capacity and has greater biological plausibility.

We believe that the definition of Robot Pain should be inspired by the nature of organisms' pain. First, the pain mechanism in organisms has evolved over thousands of years, and it is closely related to physical injury (Bagnato et al., 2015). Broom proposed that pain is the neural activity at the brain level that accompanies physical injury (Broom, 2001). This neural activity occurs primarily in the anterior cingulate cortex (ACC; Frankland and Teixeira, 2005). In addition, this neural activity can be associated with injury-related cues, such as scenes of physical injury or a dangerous object. When a similar cue occurs, the brain will generate the same neural activity and avoid potential injury in time (Karsdorp and Vlaeyen, 2009). This phenomenon is called pain memory (Eich et al., 1985). Therefore, pain is a passive response to actual physical injury and active response to potential physical injury (Sur and Amor, 2017). We argue that the Robot Pain should be designed in line with the neural mechanism of pain mentioned above—it should first respond to actual machine injury and then respond to potential machine injury.

For the cognitive process of actual physical injury, we take inspiration from the brain's Free Energy Principle (FEP; Friston, 2010). FEP proposes that the brain constantly makes predictions about the outside world, and all senses will receive real sensory information from the external world. If the predictions are consistent with the real sensory information, the brain is in a low-entropy state; if the predictions are inconsistent with the real sensory information, the brain is in a high-entropy state (Friston et al., 2006; Karl, 2012). Physical injury and other unexpected phenomena all belong to the high-entropy state (Peters et al., 2017), which indicates that the brain's predictions and sensations are inconsistent at a certain level. As the main brain region associated with pain, the ACC has also been confirmed to be associated with prediction error of different levels by multiple neuroscience studies (Oliveira et al., 2008; Castellar et al., 2010; Jessup et al., 2010). Inspired by the FEP and ACC function, we summarize a functional connection map of brain regions for cognizing actual physical injury and generating pain experience. In addition, we explored the cognitive processes for potential physical injuries. The experience of pain can be associated with injury-related cues, such as scenes or sounds of injury. The visual or auditory cortex of the brain fires accordingly and establishes synaptic connections with the ACC through associative learning. When a similar cue reappears, the brain uses these synaptic connections to generate a rapid pain experience and avoid potential injury (Wiech and Tracey, 2013). We simulated the pain-related mechanisms mentioned above with a Spiking Neural Network (SNN), and experimentally validated our model on a real robot.

This paper proposed a Brain-inspired Robot Pain Spiking Neural Network (BRP-SNN) model inspired by the neural mechanisms of organisms' pain. This model has three major contributions: (1) We summarized a neural mechanism of pain emergence from the perspective of pain evolution and the brain's Free Energy Principle (FEP). (2) We use an SNN to build a Brain-inspired Robot Pain model that can respond to both actual and potential injury, and obtain a result curve similar to the neuroscience experiment. (3) We apply the proposed model to the robot and complete the alerting actual injury task and the preventing potential injury task.

## 2. Materials and methods

### 2.1. The neural mechanism of pain

Most of the existing neuroscience literature describes the neural mechanism of pain: nociceptive stimuli specifically activate nociceptors in the skin, and nociceptive information is transmitted through the spinal cord, thalamus, and brainstem to the Anterior Cingulate Cortex (ACC) and other areas of the cerebral cortex, forming a painful experience. It is a specific pathway that has evolved over thousands of years of evolution (Sneddon, 2019). However, this pathway is not sufficient to support the modeling of Robot Pain.

Broom (2001) proposed that the emergence of pain was first related to body injury during evolution. Pain is a neural activity accompanying the body injury, and it is preserved and internalized in the brain because of its survival benefits. Nociceptors have also evolved (Perl and Kruger, 1996). In further learning, corresponding brain regions (e.g., visual and auditory cortex) will capture cues related to the injury when the body injury actually occurs. These brain regions will be connected to pain regions through associative learning (Schlund et al., 2011; Wiech and Tracey, 2013). This connection ensures a rapid avoidance response to a potential body injury.

The first step of pain emergence is the cognition of the actual body injury. The brain's Free Energy Principle (FEP) proposes that organisms are constantly maintaining the low-entropy state, and abnormal phenomena are the high-entropy state (Friston et al., 2006; Karl, 2012; Peters et al., 2017), such as body injury. Entropy cannot be quantified, but Free Energy (FE) can be approximately equal to entropy. Therefore, the FEP can be seen as the theoretical basis for cognitive actual body injury. Bogacz describes the details of the FEP and gives a formula derivation (Bogacz, 2017): the brain can never directly obtain the real state ϕ of the external world, but can only constantly estimate the external world state $\hat{\phi }$, and the body's senses can receive real sensory information *s* about the real external world state and use it to verify brain's estimate. FE in Bogacz (2017) is simplified as a negative logarithm of the joint probability distribution of external state estimation and real sensory information $-\ln\;p\left(s,\hat{\phi }\right)$, which means that the greater the joint probability distribution, the more accurate the estimate, the smaller the FE, and the smaller the entropy of the organism. The FE expression is expanded with the Bayesian formula $p\left(s,\hat{\phi }\right)=p\left(s|\hat{\phi }\right)p\left(\hat{\phi }\right)$:


(1) $$\begin{array}{l} \begin{array}{c} FE=-\ln\;p\left(s,\hat{\phi }\right)\\ \;=-\ln\;p\left(s|\hat{\phi }\right)-\ln\;p\left(\hat{\phi }\right) \end{array} \end{array}$$

The probability distribution is replaced with a Gaussian distribution *f*[*s*; *g*(.), σ] with mean *g*(.) and variance σ, supposing σ = 1:


(2) $$\begin{array}{l} FE=-\ln f_{1}(s;g_{s}(\hat{\phi }),\sigma_{s})-\ln f_{2}(\hat{\phi };\mu_{\phi },\sigma_{\phi })\\ \;=\frac{{\left[s-g_{s}(\hat{\phi })\right]}^{2}}{2\sigma_{s}}+\frac{{\left[\hat{\phi }-\mu_{\phi }\right]}^{2}}{2\sigma_{\phi }}-\frac{1}{2}\ln\sigma_{s}-\frac{1}{2}\ln\sigma_{\phi }\\ \;=\frac{1}{2}{\left[s-g_{s}(\hat{\phi })\right]}^{2}+\frac{1}{2}{\left[\hat{\phi }-\mu_{\phi }\right]}^{2} \end{array}$$

For the final derived expression, the first item represents the prediction error, where *g*_*s*_() is a generative function representing the mapping between world states and sensory information that needs to be learned empirically in advance. The brain actively estimates the state of the world $\hat{\phi }$ and predicts the sensory information through the generation function $g_{s}\left(\hat{\phi }\right)$, and the senses receive the real sensory information *s*, subtracting the two parts can produce prediction error. The second item represents the prior error, which indicates that the brain's estimates must be compared with the prior knowledge μ_ϕ_ stored in the brain. Prediction error and prior error together determine the value of the FE. The FE has three main functions: 1. It can be used to guide the brain to change its estimates. 2. It can be used to guide the brain to perform actions. 3. As an internal variable, it can reflect the internal state of the brain and can be used to study a variety of cognitive functions.

The cognitive process of body injury and pain emergence can be explained by FEP: The brain can predict the body sensory information of all modalities [*s*_1_, *s*_2_, ..., *s*_*n*_] based on the current body state $\hat{\phi }$. This body state can be known based on prior knowledge stored by the brain (e.g., the previous moment's body state ϕ′ and the performed action *a*′). Equation (3) shows the calculated rule of the FE in this scenario. If FE is 0, it means that the body is in a normal state. If FE is >0, it means that the prediction error arises and the body is in an injured state, which leads to the pain experience.


(3) $$\begin{array}{l} \begin{array}{c} FE={\left[s_{1}-g_{s_{1}}\left(\hat{\phi }\right)\right]}^{2}+{\left[s_{2}-g_{s_{2}}\left(\hat{\phi }\right)\right]}^{2}+...\\ +{\left[s_{n}-g_{s_{n}}\left(\hat{\phi }\right)\right]}^{2}+{\left[\hat{\phi }-g_{\phi }\left(\phi^{\prime },a^{\prime }\right)\right]}^{2} \end{array} \end{array}$$

It is worth noting that the brain has many prediction processes at different levels in addition to the above prediction from body state to body sensation. In the case of an injection, the prediction of whether or not be pain is essentially an event-level prediction, which is a prediction of the pain event in the context where the pain has evolved. This is a different level from the prediction of cognitive body injury we described above.

Numerous neuroscientific studies have shown that pain is associated with the Anterior Cingulate Cortex (ACC) of the brain (Davis et al., 1997; Frankland and Teixeira, 2005; Du et al., 2020). The ACC has also been shown to be responsible for prediction error computation and conflict detection (Silvetti et al., 2013; Wiech and Tracey, 2013). Silvetti et al. proposed that the ACC contains neurons representing event prediction and neurons representing prediction errors. They argued that the ACC can receive feedback from the external environment, which together with the prediction neurons leads to the firing of the prediction error neurons (Silvetti et al., 2011). Inspired by the FEP and ACC function, we summarize the connectivity map of brain regions that cognize actual physical injuries and generate pain experiences.

**Figure 1:**
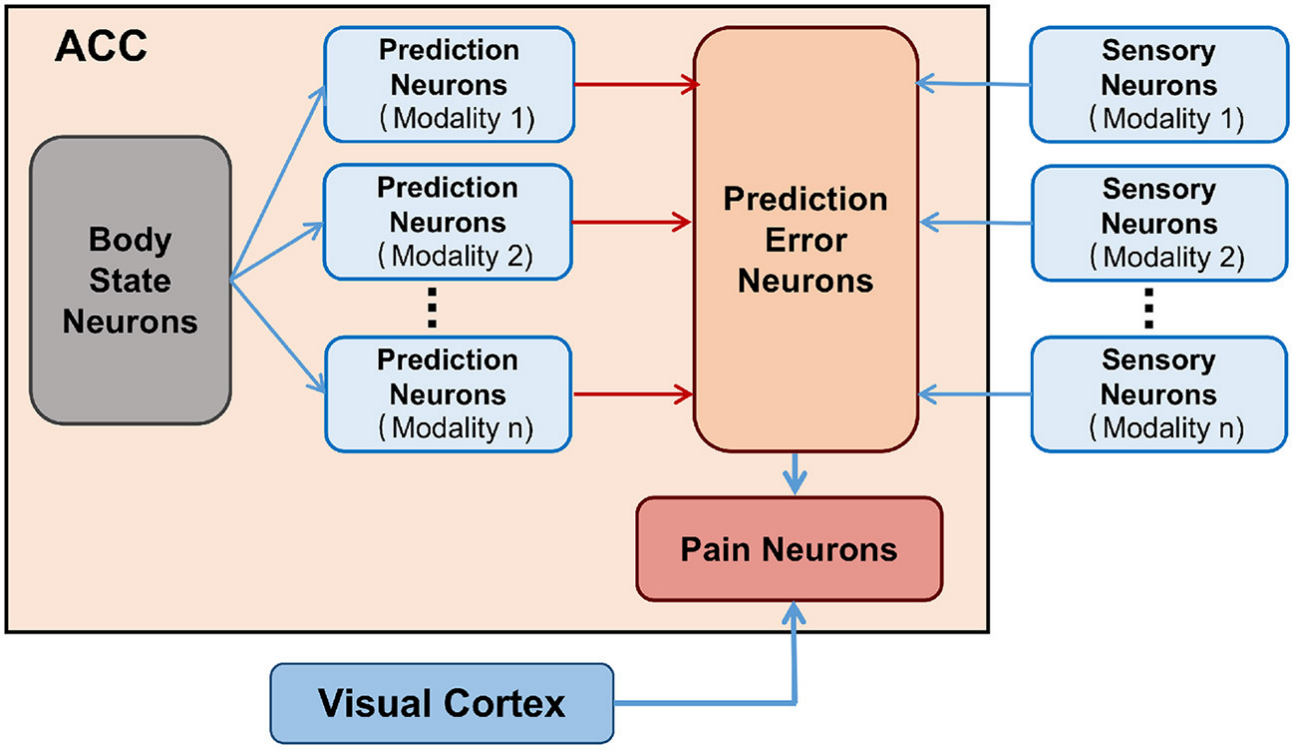
The connectivity map of brain regions of Pain. Body state neurons encode body state. Prediction neurons encode predictions of sensory information. Pain neurons characterize whether the body is in a pain state. Sensory neurons encode received real sensory information. Visual Cortex encodes injury-related cues.

As shown in Figure 1. The body state neurons of ACC constantly infer self body state and predict multi-modality sensory information, and the corresponding prediction neurons of ACC are activated. The senses of the body can receive the real sensory information and the corresponding sensory neurons are activated. When the body state is normal, the information from the sensory neurons and the prediction neurons are consistent, which does not cause the prediction error neurons of ACC to fire, and the body in a low-entropy state. When the body is injured, it definitely causes the sensory information of some modalities to be inconsistent with the predicted information, causing prediction error neurons to fire and the brain cognizes that the body is injured. Then pain neurons are activated to fire and produce pain experiences. The body in a high-entropy state. We also investigated the neural mechanisms associated with pain memory. Here we use vision as an example, as shown in the gray module in Figure 1. Visual Cortex has a direct connection to the pain-related region in the ACC (Wiech and Tracey, 2013). The Visual Cortex will capture the injury-related cues when the body injury occurs. Due to the temporal correlation, the weight connections between the corresponding visual cortex neurons and the pain-related neurons of the ACC will be enhanced by association learning. The brain will use these connections to identify potential injury and avoid it.

### 2.2. Brain-inspired robot pain spiking neural network

This subsection introduces the implementation details of the BRP-SNN inspired by the neural mechanisms of pain. We first introduce the basic tools and methods, then introduce the overall architecture of the BRP-SNN.

#### 2.2.1. Neuron model and synapse learning method

The emergence of a spiking neural network (SNN) has facilitated the development of neuromorphic computing (Yang et al., 2021). In recent years, SNNs have been successfully applied to many aspects, such as meta-learning and few-shot learning (Yang et al., 2022b,c), working memory and decision making (Zhao et al., 2018; Yang et al., 2022a). The Leaky Integrate-and-Fire (LIF) neuron is the most common neuron model for SNN (Tal and Schwartz, 1997; Gerstner and Kistler, 2002), and we use it as the basic unit of our BRP-SNN. The LIF neuron dynamics are described by the following Equations (3) and (4):


(4) $$\begin{array}{l} \tau_{m}\frac{du}{dt}=-\left[u_{t}-u_{rest}\right]+RI\left(t\right) \end{array}$$

(5) $$\begin{array}{l} \underset{\delta \to 0}{\lim}u\left(t^{f}+\delta \right)=u_{reset} \end{array}$$

*u*_*t*_ is the membrane potential of the neuron at time *t*, *u*_*rest*_ is the membrane potential at steady-state, *R* is the resistance, *I*(*t*) is input current, and τ_*m*_ is the time constant. When the membrane potential *u*_*t*_ exceeds a certain threshold *u*_*th*_, the neuron fires, and *t*_*f*_ is the firing time. Once the neuron fired, the membrane potential returns to its reset state *u*_*reset*_. In this paper, the parameters of the LIF neuron model are: *u*_*rest*_ = *u*_*reset*_ = −65*mV*, τ_*m*_ = 10*ms*, *u*_*th*_ = −50*mV*.

We use spike-timing-dependent-plasticity (STDP) as a synapse learning rule to update synaptic weights. STDP is the most basic learning method in the brain, which relies on the time difference between the firing of pre-synaptic neurons and post-synaptic neurons to train the synaptic weight (Dan and Poo, 2004; Sjöström and Gerstner, 2010; Markram et al., 2012; Chevtchenko and Ludermir, 2020). The weights update rule is written as Equation (5):


(6) $$\text{Δ}\omega =\{\begin{array}{cc} A^{+}exp(\frac{\text{Δ}t}{\tau^{+}}), & \text{Δ}t<0\\ -A^{-}exp(\frac{-\text{Δ}t}{\tau^{-}}), & \text{Δ}t>0 \end{array}$$

With Δ*t* = *t*_*i*_−*t*_*j*_, where *t*_*i*_ and *t*_*j*_ are the firing time of the pre-synaptic neuron and the post-synaptic neuron respectively. *A*^+^ and *A*^−^ are the learning rates. τ_+_ and τ_−_ are the time constants. According to this rule, the connections will be strengthened when pre-synaptic neurons fire before post-synaptic neurons and will be weakened when pre-synaptic neurons fire after post-synaptic neurons. In this paper, τ_+_ = τ_−_ = 10*ms*, *A*^+^ = 0.5, *A*^−^ = 0.1. The choice of these parameters is based on the settings in reference (Sjöström and Gerstner, 2010). The values of τ_+_ and τ_−_ are consistent with the values in that literature. The *A*^+^ and *A*^−^ reflect the strength of LTP and LTD, respectively, and the effect of LTP is considered more in this paper.

#### 2.2.2. Population coding method

Population coding is a neuron coding method of SNN. The representation information of one neuron is limited, and the population coding is more effective and biologically plausible (Fang and Zeng, 2021; Fang et al., 2021).

**Figure 2:**
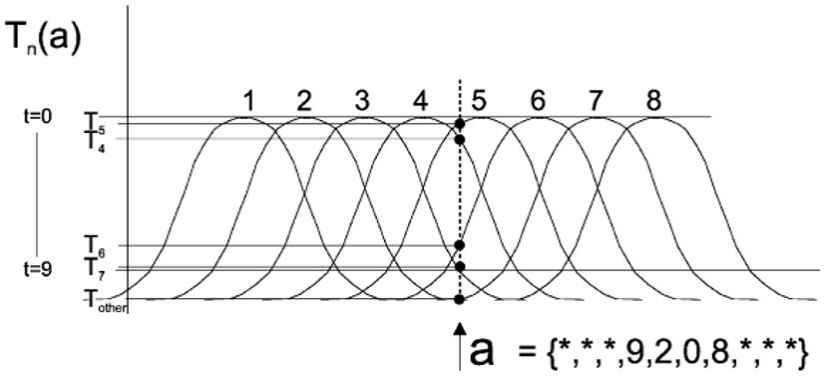
The Gaussian Receptive Field (GRF) coding method (Bohte et al., 2002).

Population coding uses a neuron population to encode input data, which can represent continuous values. In this paper, we adopted the Gaussian receptive field (GRF) population coding method mentioned in Bohte et al. (2002). Each dimensional receptive field is a group of neurons, where each neuron corresponds to a Gaussian activation function. Each input will cause all neurons in the receptive field to fire at different times through a different Gaussian activation function, Figure 2 shows eight neurons per dimensional receptive field. For each input pattern, the response time *t* is calculated for each neuron, and neurons with response times >9 are coded as not triggered because they are considered insufficiently excitable. The single input *a* = {*, *, *, 9, 2, 0, 8, *, *} was encoded by eight Gaussian activation functions, and the black dot marking *T*_*i*_ indicates the firing time of the i-th neuron involved in its encoding.

#### 2.2.3. Model implementation

When body states are known, the prediction error generated by the prediction process from the body state to body sensations of all modalities can lead to pain. According to this mechanism, the specific cognitive process of Robot Pain is as follows: the robot continuously infers the current body state through the previous moment state and the executed action, and predicts the sensory information that will be received by all robot sensors. When the prediction error occurs, it indicates body injury, which in turn leads to Robot Pain.

**Figure 3:**
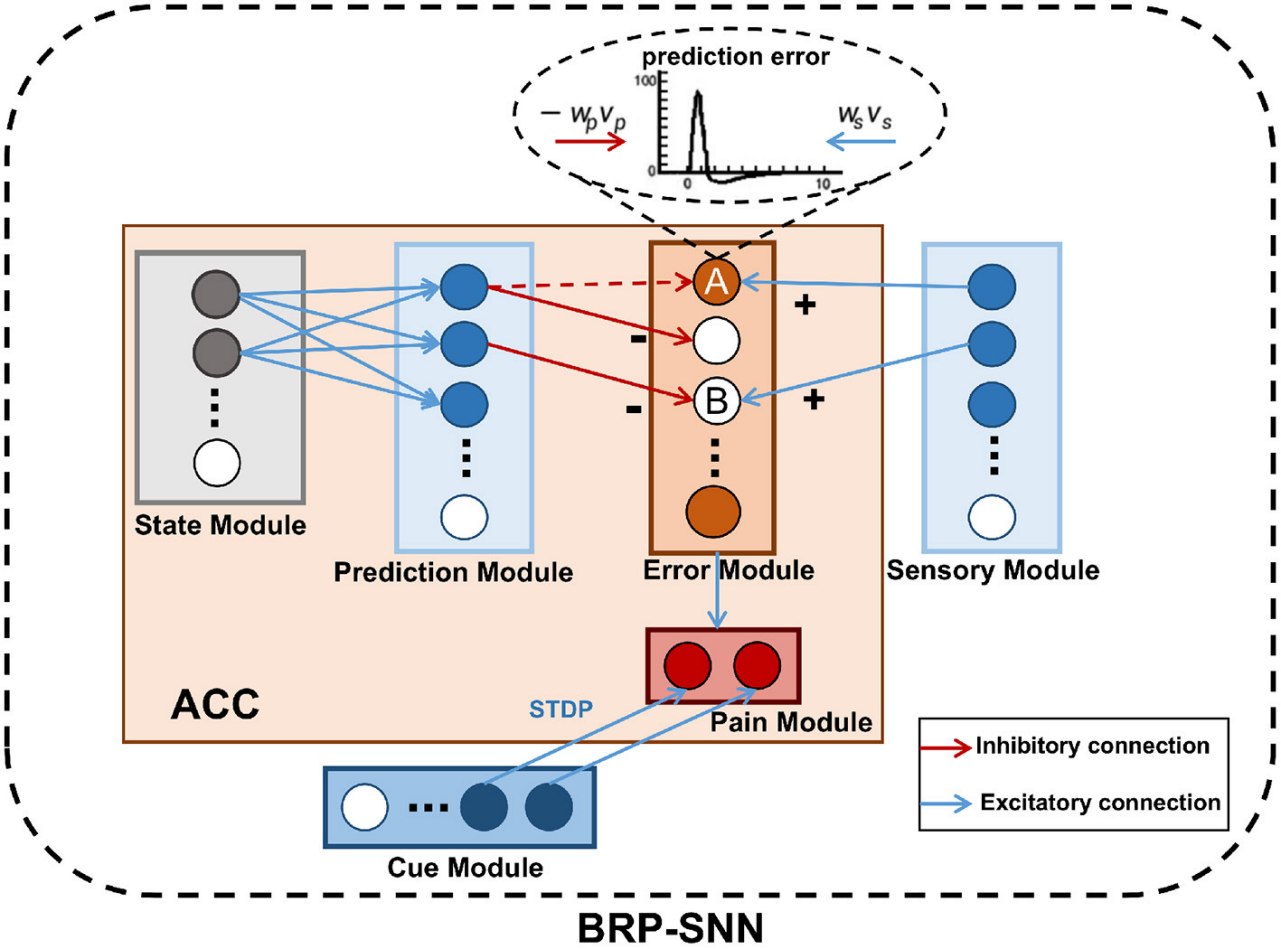
The network architecture of the BRP-SNN. The State Module can activate the corresponding Prediction Module through forwarding prediction. The Prediction Module to the Error Module is the excitatory connection, and the Sensory Module to the Error Module is the inhibitory connection. The Error Module represents prediction error. The Pain Module represents whether Robot Pain is produced or not. The Cue Module encodes the injury-related cues.

As shown in Figure 3, The BRP-SNN simulates the function and connection of the brain regions mentioned in Figure 1, which contains the State Module, Prediction Module, Sensory Module, Error Module, Pain Module, and Cue Module. Each module is a neuron population. The State Module encodes different body states. The Prediction Module represents the predictive sensory information of different modalities. The Sensory Module encodes the real sensory information of different modalities. The Error Module represents prediction error. The Pain Module represents whether Robot Pain is produced or not. The Cue Module encodes the injury-related cues. The blue arrows in Figure 3 indicate excitatory connections, and the red arrows in Figure 3 indicate inhibitory connections.

The mapping relationship between the State Module and the Prediction Module needs to be learned empirically by collecting robot body data. The connection weights are established through STDP in the process of learning. After learning, the State Module can activate the corresponding Prediction Module through forwarding prediction. The Error Module represents the prediction error, once any neuron in this module fires, indicating that the prediction error arises. According to the excitatory and inhibitory synaptic connections in the ACC mentioned in Silvetti et al. (2011), we designed that the Prediction Module to the Error Module is the inhibitory connection, and the Sensory Module to the Error Module is the excitatory connection. The dynamics of neurons in the Error Module are described by Equation (6):


(7) $$\begin{array}{l} \text{Δ}u=-\omega_{p}v_{p}+\omega_{s}v_{s}\left(\omega_{p},\omega_{s}>0\right) \end{array}$$

Δ*u* represents an update of the membrane potential of the neurons in the Pain Module. −ω_*p*_ represents inhibitory weights. ω_*s*_ represents excitatory weights. *v*_*p*_ is the input from the Prediction Module. *v*_*s*_ is the input from the Sensory Module. When the firing pattern of the Prediction Module is consistent with the Sensory Module, the effects of excitatory and inhibitory on the neuron of the Error Module are counteracted, and the Error Module does not fire, such as the B neuron in Figure 3, indicating a normal body state. When the firing pattern of the Prediction Module is inconsistent with the Sensory Module, the effects of excitatory and inhibitory are not counteracted and specific neurons in the Error Module will fire. For example, the excitatory effect from the Sensory Module acts on A neuron, but the inhibitory effect from the Prediction Module acts on the other neuron. The red dashed arrow in Figure 3 indicates an inhibitory effect that has not yet been established. A neuron receives only excitatory effects, so it fires. The Error Module to the Pain Module is excitatory and fully connected. When the robot body state is known, any neuron fire of the Error Module causes the Pain Module to fire, indicating the Robot Pain state.

The Cue Module encodes the injury-related cues, such as a dangerous object captured by the camera. The connections between the Cue Module and Pain Module can be established through STDP by association learning. This BRP-SNN can not only recognize actual machine injury and produce Robot Pain, but also recognize injury-related cues and avoid potential machine injury.

## 3. Experiment

### 3.1. Experiment settings

We use the Nao robot as an experimental platform and apply the BRP-SNN to implement two tasks: the alerting actual injury task and the preventing potential injury task.

**Figure 4:**
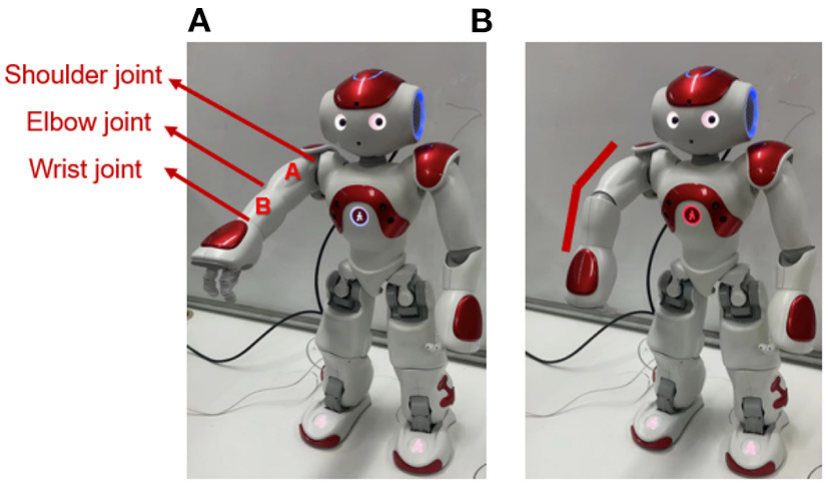
The Nao robot. **(A)** Shows the assumed normal body state. **(B)** Shows the assumed injured state.

As shown in Figure 4A, the left arm of the Nao robot contains three joints: shoulder joint, elbow joint, and wrist joint, and contains two links: link A and link B. In order to mimic the robot injury process without actually damaging the robot. Our experiments assume that the left arm is in a normal body state when it is straight and in an injured state when it is bent. This means that it is assumed that link A and link B are an integral link. The elbow joint sensor (between linkA and linkB) will not receive any action command from the robot, and the robot will not process any input information from this elbow joint sensor. Under this assumption, the bending elbow as shown in Figure 4B is an undesirable deformation of the robot that is similar to a human fracture.

In this experiment, we only consider two sensory modalities of the robot: proprioception *s*_*p*_ and vision *s*_*v*_, which were the angle data of each joint of the arm and the position coordinate data of the hand in the camera. As in Equation (8), a special case of Equation (3).


(8) $$\begin{array}{l} \begin{array}{c} FE={\left[s_{v}-g_{s_{v}}\left(\hat{\phi }\right)\right]}^{2}+{\left[s_{p}-g_{s_{p}}\left(\hat{\phi }\right)\right]}^{2}+{\left[\hat{\phi }-g_{\phi }\left(\phi^{\prime },a^{\prime }\right)\right]}^{2} \end{array} \end{array}$$

The State Module encodes the body state. Lanillos and Cheng (2018) propose that the robot should determine body state through multi-modal sensations using the FEP, corresponding to the FE's function1 described in Section 2.1. They set the body state as a variable that requires continuous learning. The robot will actively estimate the current body state and verify this estimate by sensory information received from sensors, continuously updating this estimate until the prediction error (FE) is zero, to determine the current body state. Inspired by this, we divide our experiment into two steps: Step 1 is to determine the robot's initial body state using the method proposed by Lanillos and Cheng (2018). This corresponds to the FE's function 1. Step 2 is to infer the body state at each moment by the initial state and the executed actions during the task, predict each sensation, and calculate the prediction error at each moment, to determine whether the robot is in the injury state. This corresponds to the FE's function 3. Step 1 uses the State Module, Prediction Module, Sensory Module, and Error Module of BRP-SNN. Step 2 uses the State Module, Prediction Module, Sensory Module, Error Module, and Pain Module of BPR-SNN.

The body state is a high-dimensional variable in the brain. For computational purposes, Lanillos et al. defined body state in the proprioception data space. This does not mean that body state is equivalent to proprioception, but only consistent with its data dimension and the range of values. We follow this assumption that body states are defined in the proprioception data space and encoded by the State Module of BRP-SNN. The Sensory Module encodes real proprioception and vision data. We set the initial value of the body state and continuously make predictions to both sensor data. The prediction error (Error Module fires) will guide the body state estimation update using the exhaustive search algorithm until the Error Module no longer fires. After the initial robot body state is determined, the subsequent body states can be inferred by prior knowledge (the body state and the executed actions at the previous moment). In a normal situation, there is no prediction error when the current body state makes predictions about the current sensation, the Error Module does not fire. When the body is injured, the Error Module fires, which in turn causes the Pain Module fires, and the robot is in a pain state.

**Figure 5:**
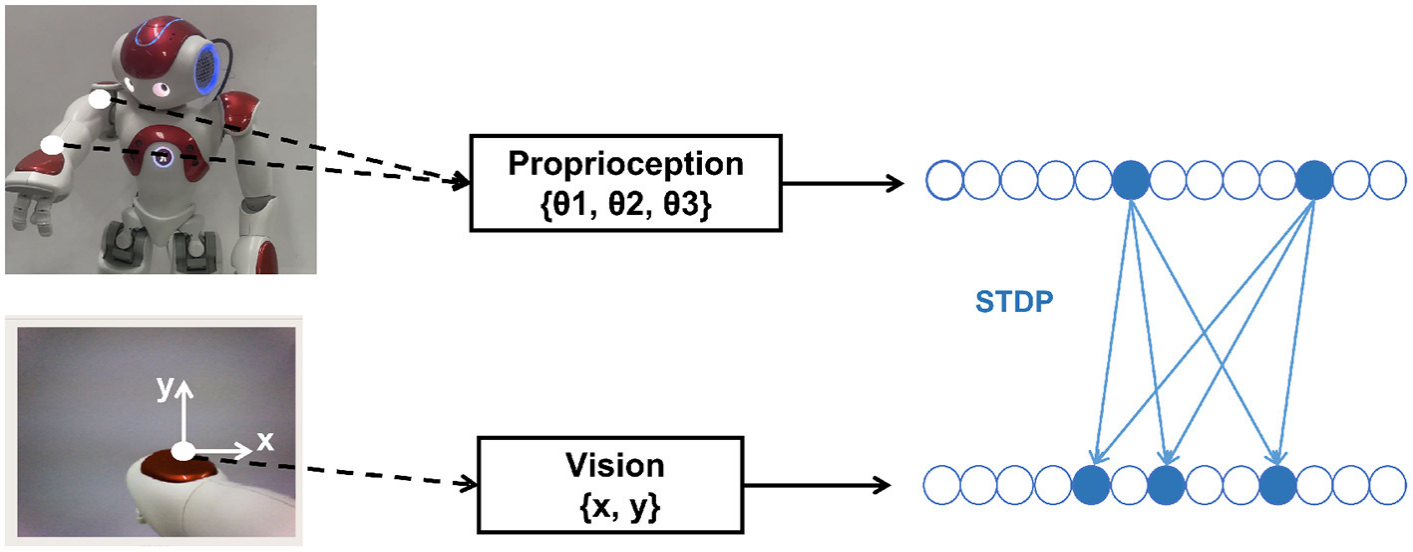
Collecting dataset and training by STDP. Proprioception data only includes the ShoulderPitch joint angle θ_1_, the ShoulderRoll joint angle θ_2_, and the WristYaw joint angle θ_3_, and the Elbow joint is not considered. Vision data is the coordinate position (*x, y*) of the hand in the camera.

The mapping relationship between body state and proprioception *g*_*s*_*p*__() and vision *g*_*s*_*v*__() should be learned in advance for the forwarding prediction of the State Module to the Prediction Module. The robot collects training data through several random movements (motor babbling) while the left arm is straight. As shown in Figure 5. Proprioception data only includes the ShoulderPitch joint angle θ_1_, the ShoulderRoll joint angle θ_2_, and the WristYaw joint angle θ_3_, and the Elbow joint is not considered. Vision data is the coordinate position (*x, y*) of the hand in the camera, obtained by grayscale processing of the image captured by the camera and calculating the median value of the corresponding pixel coordinates. The body stateϕ is set in the proprioception data space, so the mapping relationship *g*_*s*_*p*__(ϕ) = ϕ. The mapping relationship *g*_*s*_*v*__() needs to be learned using STDP.

### 3.2. The alerting actual injury task

#### 3.2.1. Experiment results

We designed two actual injury paradigms in the alerting actual injury task: undesirable body deformation and motor system injury.

**Figure 6:**
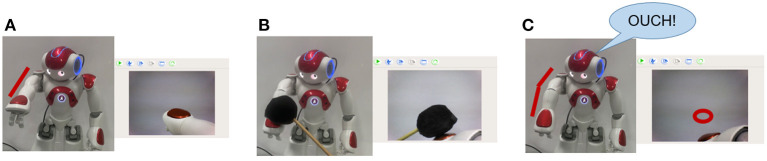
The Alerting Actual Injury Task (the assumed undesirable body deformation). **(A)** Shows the normal state of the robot. **(B)** Shows that the elbow is bent with a black object. **(C)** Shows that the robot is in the Robot Pain state and makes an alarm, and the red circle represents the predicted position of the hand by BRP-SNN.

In the case of undesirable body deformation, when the elbow bending caused by a black object occurs, the robot's prediction will be inconsistent with the real sensory data. Then the robot turns into the Robot Pain state and says “OUCH!” for alarm. Figure 6A shows the normal state of the robot. Figure 6B shows that the elbow is bent with a black object. Figure 6C shows that the robot is in the Robot Pain state and makes an alarm, and the red circle represents the predicted position of the hand by BRP-SNN.

**Figure 7:**
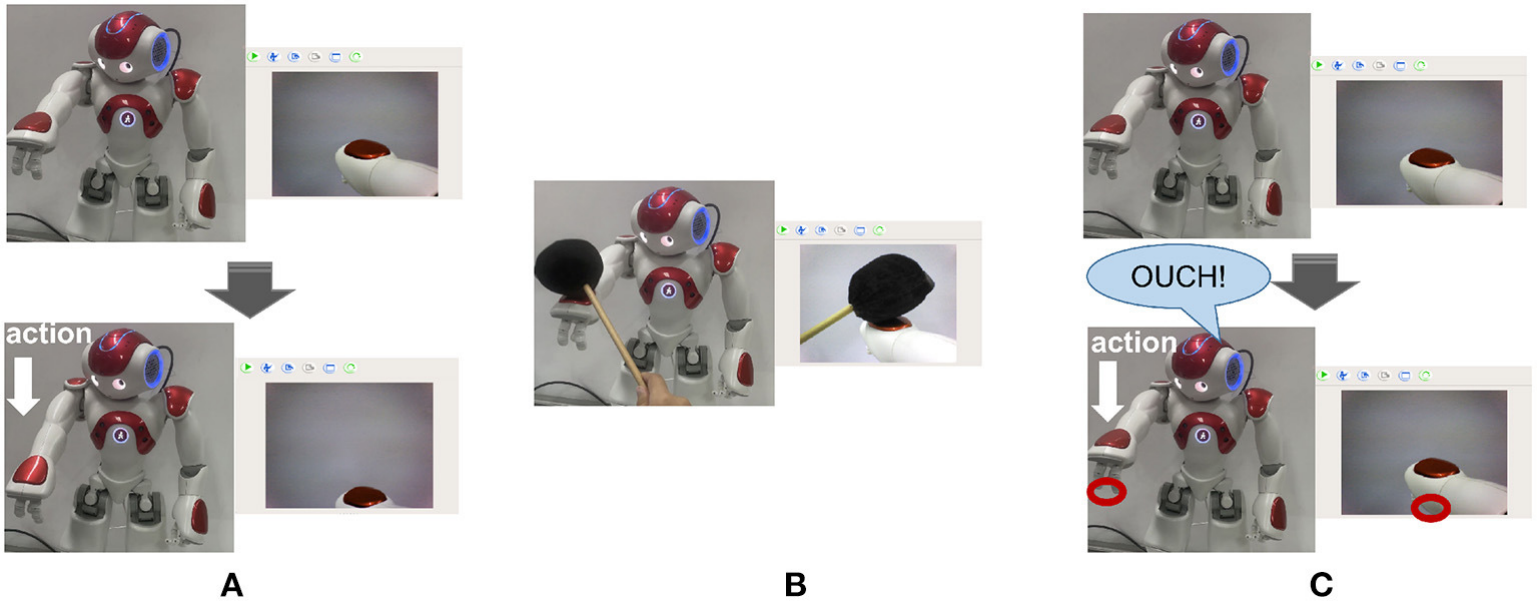
The Alerting Actual Injury Task (the assumed motor system injury). **(A)** Shows the normal state of the robot. The hand's position in the camera should also move downward when performing the downward action. **(B)** Shows hitting the arm with a black object, **(C)** shows that the motor system injury occurs and the position of the hand will not move. The robot is in the Robot Pain state and makes an alarm, and the red circle represents the predicted position of the hand by BRP-SNN.

In the case of motor system injury, we made the following assumption: when a black object hits the robot's arm and touches the tactile sensor, the robot does not execute any action at the program level, simulating motor system injury. In this experiment, the robot constantly does random movements and predicts the sensory information at the next moment by its current body state and the action command (prior knowledge). When the injury occurs, the predicted position is inconsistent with the real sensory. Then the robot is in the Robot Pain state and says “OUCH!” for alarm. Figure 7A shows the normal state of the robot. The hand's position in the camera should also move downward when performing the downward action. Figure 7B shows hitting the arm with a black object, Figure 7C shows that the motor system injury occurs and the position of the hand will not move. The robot is in a Robot Pain state and makes an alarm, and the red circle represents the predicted position of the hand by BRP-SNN.

#### 3.2.2. Model analysis

**Figure 8:**
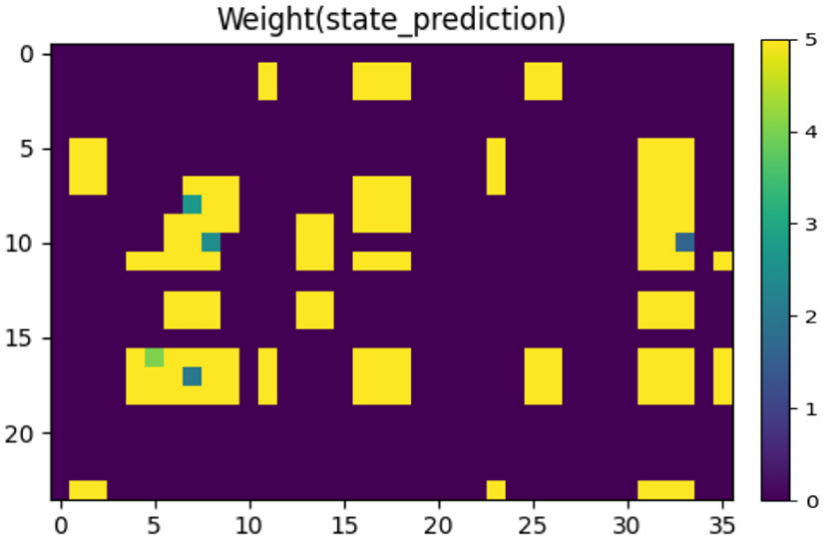
The synaptic weights between the State Module and the neurons that characterize vision information in the Prediction Module. The closer the color is to yellow, the larger the weight is; and the closer the color is to purple, the smaller the weight is.

Figure 8 shows the synaptic weights between the State Module and the neurons that characterize vision information in the Prediction Module, indicating the mapping relationship between the body state and the prediction of vision information. These weights are learned by STDP training rules from the data set collected in advance (as shown in Figure 5). The closer the color is to yellow, the larger the weight is; and the closer the color is to purple, the smaller the weight is.

**Figure 9:**
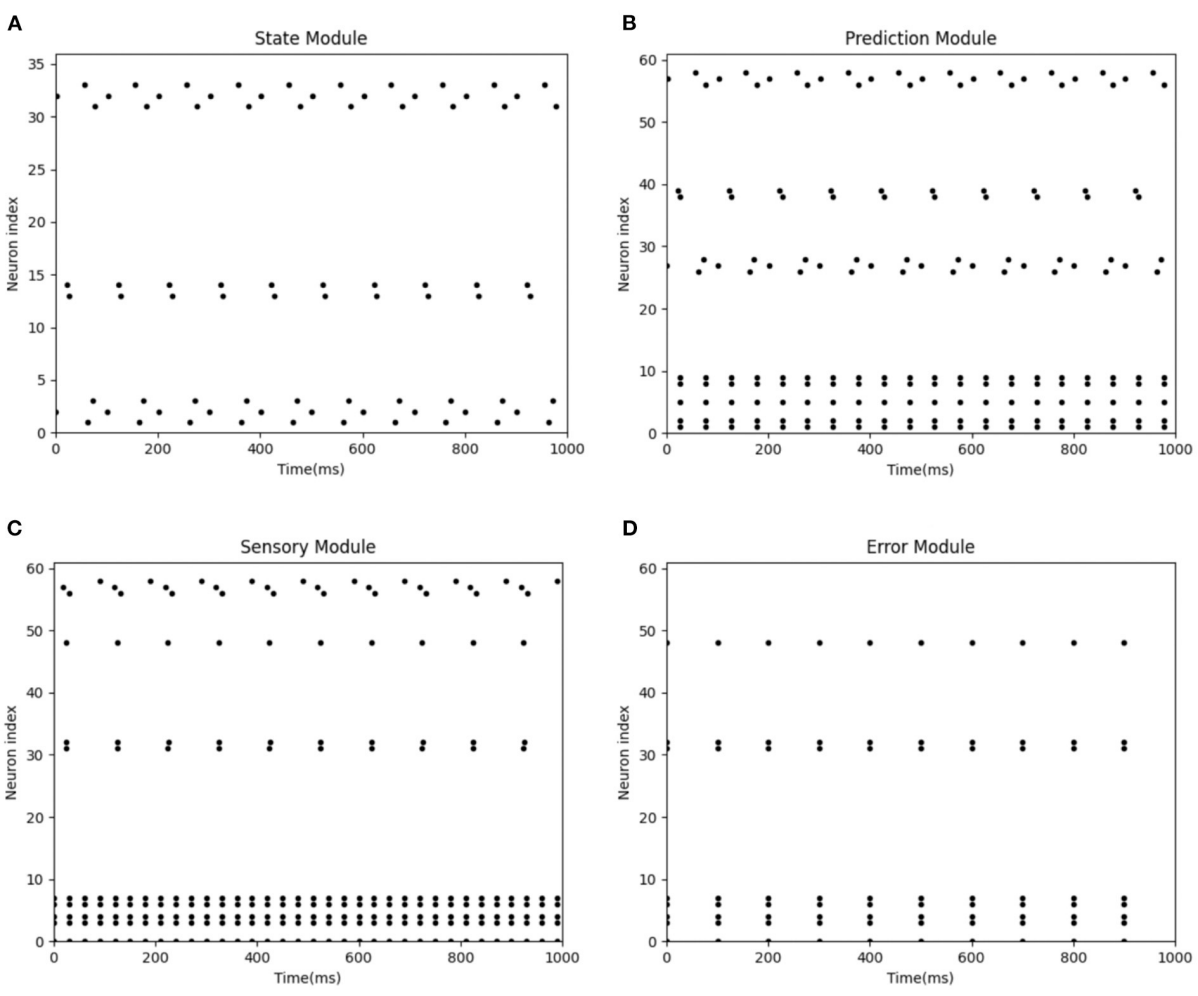
The spike diagrams of the BRP-SNN in the actual injury task. The X-axis represents the time, and the Y-axis represents the neuron index. **(A)** Represents the firing pattern of the State Module. **(B)** Represents the firing pattern of the Prediction Module when predicting sensory information. **(C)** Represents the firing pattern of the Sensory Module when receiving the real sensory information. **(D)** Shows the firing pattern of the Error Module.

Figure 9 represents the spike diagram of modules of the BRP-SNN in the actual injury condition. The X-axis represents the time, and the Y-axis represents the neuron index. Figure 9A represents the firing pattern of the State Module. Figure 9B represents the firing pattern of the Prediction Module when predicting sensory information. Figure 9C represents the firing pattern of the Sensory Module when receiving the real sensory information. It is shown that the firing neuron index of the Prediction Module is inconsistent with the Sensory Module, so the Error Module fries. Figure 9D shows the firing pattern of the Error Module. Due to the excitatory connection between the Error Module and the Pain Module, the Pain Module then fires, indicating the Robot Pain state. In BRP-SNN, we design the time step as 100 ms, and the robot collects sensor information per 100 ms. Figure 9 represents the firing of each module during 1,000 ms when the robot experiences actual injury. The sensor acquires abnormal sensory information every 100 ms, which is encoded by the Sensory Module, which causes the Error Module to fire. The Error Module is fired 10 times in 1,000 ms.

**Figure 10:**
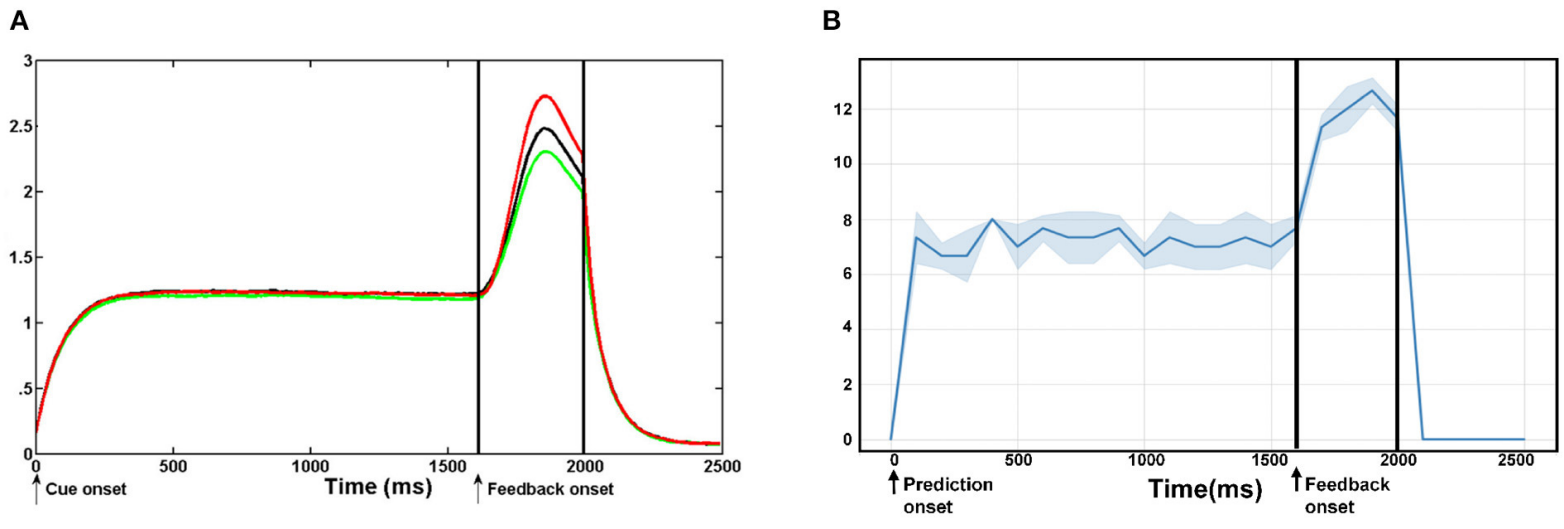
The comparative analysis of our proposed model with the previous neuroscience model–RVPM. **(A)** Shows the average activation of the whole ACC at the RVPM under different levels of reward events. **(B)** Shows the average firing rate of the whole ACC module of the BRN-SNN. This result shows that our BRP-SNN has the same change trend as RVPM under the same experimental conditions.

Figure 10 shows the comparative analysis of our proposed model with previous neuroscience studies about ACC. Silvetti et al. (2011, 2013) proposed a neural model of the ACC–the Reward Value and Prediction Model (RVPM) and validated it in model-based human fMRI experiments. Figure 10A represents the average activation intensity of the ACC module of the RVPM over time. In this experiment, the cue signal is first fed into the model, which is a square wave with a 2,000 ms duration and unit amplitude. Then, a prediction process with a 2,000 ms duration starts. The reward feedback signal with a 400 ms duration is added at 1,600 ms. Each experiment is 2,500 ms and is performed 20 times. We simulated the same experiment steps in the BRP-SNN. As shown in Figure 10B, body state information (robot proprioceptive information) was first fed to the State Module to start a prediction process with a 2,000 ms duration. Then, a feedback signal (real visual information) of 400 ms duration is added to the Sensory Module at 1,600 ms. The total duration of the experiment is also 2,500 ms. We performed it 20 times and calculated the average activation intensity of the whole ACC. It is worth noting that each unit of RVPM represents a continuous value, and the calculation rule for the average activation intensity is the sum of the amplitudes of multiple signals. However, each module of our BRP-SNN is a neuron group with discrete spike signals, and the calculation rule for the average activation intensity is the sum of the firing rates of all modules in ACC every 100 ms. The different data morphology and calculation rules result in inconsistent limits on the Y-axis in the two figures. But, both figures reflect the same change trend of average activation intensity: At the beginning of the prediction, both curves increase significantly, indicating that the prediction module is firing. At the reception of an environmental feedback signal that is inconsistent with the prediction, both curves increase again, indicating that the prediction error module is firing. At the end of the prediction, both curves decrease to 0, indicating that the whole ACC is at rest state. This result indicates that our BRP-SNN has the same change trend as RVPM under the same experimental conditions.

### 3.3. The preventing potential injury task

#### 3.3.1. Experiment results

**Figure 11:**
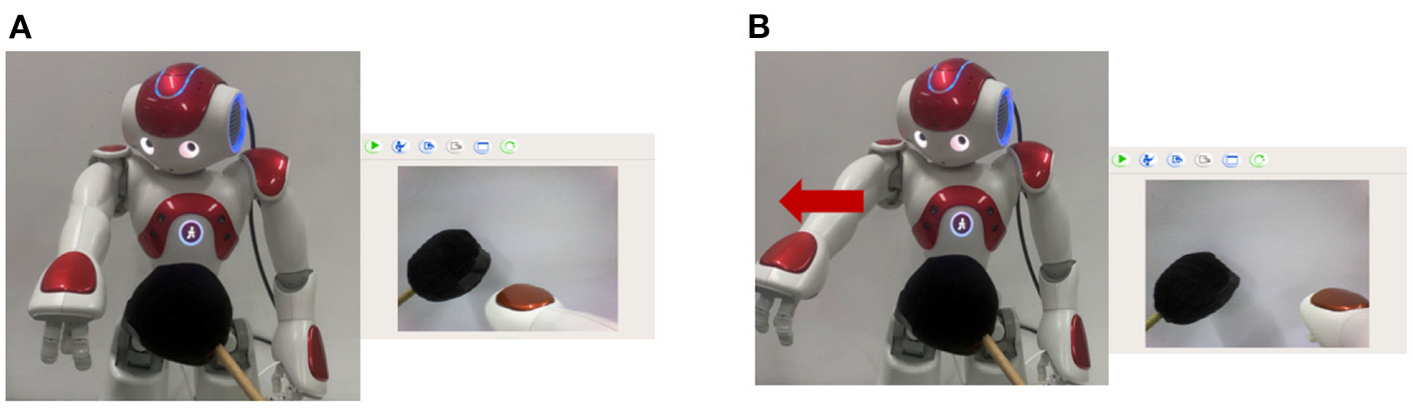
The preventing potential injury task. **(A)** Shows that the dangerous black object approaches the robot, and the robot recognizes it. **(B)** Shows that the robot executes avoidance behavior to prevent potential injury.

In both actual injury paradigms, the black object is captured by the camera and encoded by the Cue Module, and establishes the connection between the Cue Module and the Pain Module by association learning. When the robot sees the black object again, even if the actual injury has not occurred, the Pain Module also fires and then executes the avoidance behavior. Figure 11A shows that the dangerous black object approaches the robot, and the robot recognizes it. Figure 11B shows that the robot executes avoidance behavior to prevent potential injury.

#### 3.3.2. Model analysis

**Figure 12:**
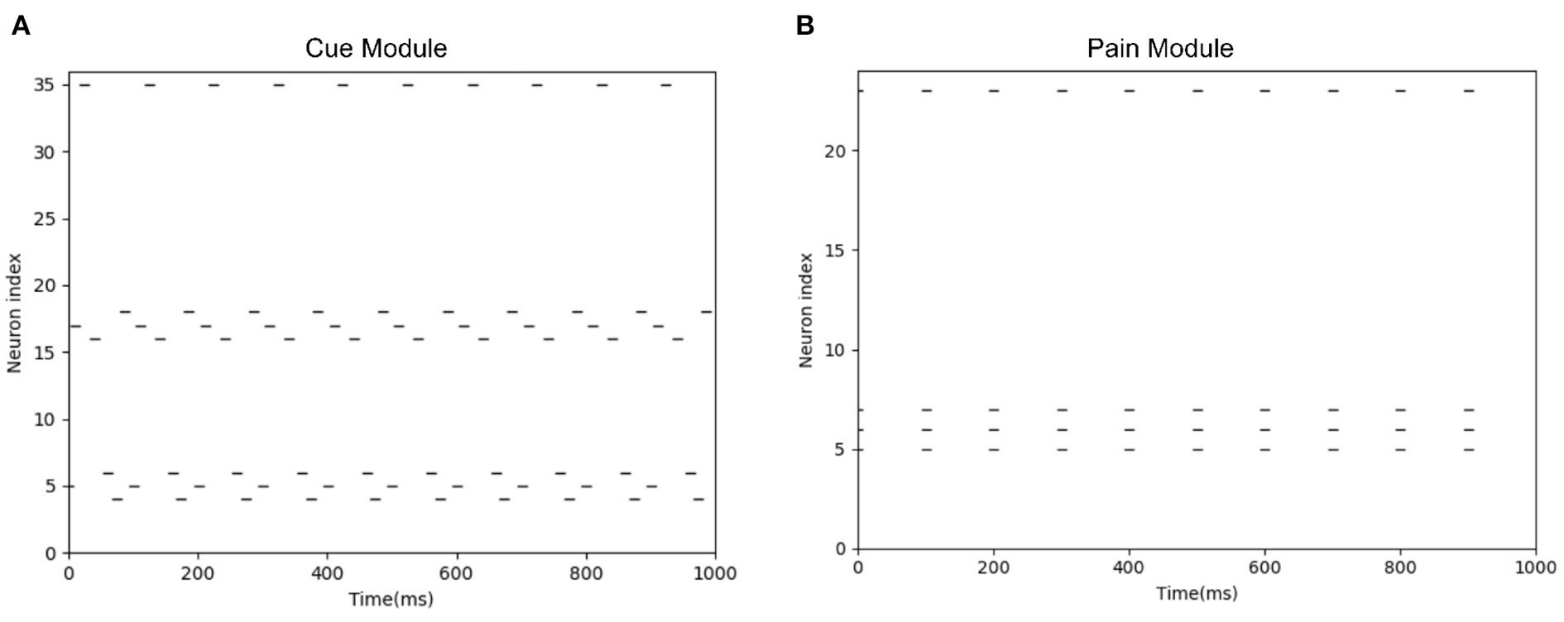
The spike diagrams of the BRP-SNN in the potential injury task. The X-axis represents the time, and the Y-axis represents the neuron index. **(A)** Represents the firing pattern of the Cue Module. **(B)** Represents the firing pattern of the Pain Module.

When the camera of the robot detects the black object, this black object is encoded by the Cue Module. When the actual injury occurs, the connection between the Cue Module and the Pain Module can be established. When the robot sees the black object again, the Cue Module fire, as shown in Figure 12A. Then the Pain Module fire, as shown in Figure 12B, and avoid potential injury.

## 4. Discussion

This paper proposes a Brain-Inspired Robot Pain model that simulates the neural mechanism and behavioral results of organisms' pain. This model can alert to actual injury and avoid potential injury, and obtain the result curves similar to previous neuroscience experiments.

In this paper, we argue that pain is a kind of neural activity occurring at the brain level accompanied by physical injury, and it is a subjective reflection of the objective physical injury event. It means that the organism has a self-body model, which reflects the coupling relationship between the multi-modality information of the body, such as the multisensory modalities and motor modality. Once a physical injury event occurs, the coupling relationship will be broken, and the organism will be in a state of increased entropy. Then the brain will perceive this abnormal state and react to it, gradually evolving into pain. In our work, only two sensory modalities and one motion modality of the robot are considered. If robots have more sensors in the future, our model can also be generalized to more modalities.

We have made certain assumptions in robot experiments, such as taking normal elbow bending as an abnormal deformation of robot and designing motor system injury at the program level. That is because we cannot actually harm the robots in the lab. The two tasks of the robot are only a validation of our BRP-SNN model. We also hope to use the principle of this model to make the robots produce 'Robot Pain' in a real work environment, to realize the alarm and prevention of injury in the future.

In the training phase of the connection weights between the State Module and the Prediction Module, we set the initial weights to 0 and use the STDP to adjust the weights according to the correlation of the firing times of the pre-synaptic and post-synaptic spikes. In this paper, we mainly emphasize the effect of LTP, and the weight values that meet the LTP condition will be increased. We manually restricted the size of the weights to the range [0, 5] to facilitate observation and debugging.

There are still some limitations to our work. First, our model implements the task on a robot under the specific conditions we designed, so its robustness has not been verified yet. In future work, we will validate the utility of our model under other experimental conditions (e.g., robot data from other modalities). Second, in the preventing potential injury task, the process from the firing of the Cue Module (potential danger detection) to triggering the firing of the Pain Module (pain state activation) is trained by STDP through association learning. However, the transition from the pain state activation to the execution of the avoidance action is artificially set by us. In fact, for an organism, the avoidance action is learned by trial and error. In the next step, we will explore the learning process of pain-triggered self-defensive actions and the relationship between pain models and intrinsic rewards in the reinforcement learning method. In addition, we plan to further investigate more neural mechanisms and computational models of cognitive functions related to pain, such as pain empathy. Pain empathy is an important factor to promote harmonious coexistence among social groups, and robots with empathic abilities will be more moral (Asada et al., 2009; Asada, 2015). In the future, we will explore the computational models of pain empathy and the altruistic behavior of robots.

This paper proposes a Brain-Inspired Robot Pain Spiking Neural Network inspired by the neural mechanism of organisms' pain, enabling the robot to have a human-like pain capacity. We explored the neural mechanism of pain emergence from the perspective of pain evolution and the brain's Free Energy Principle, and we used SNN to simulate relevant brain regions' functions and connections to build a Robot Pain model with the STDP method and the population coding method. Our model is inspired by the pain's neural mechanisms and achieves not only the alarming of actual machine injuries but also the prevention of potential danger, which has positive implications for the integration of pain concepts into the robotics field. Our work is a meaningful step toward creating more brain-like intelligent robots in the future.

## Data availability statement

The raw data supporting the conclusions of this article will be made available by the authors, without undue reservation.

## Author contributions

HF and YZ designed the study, developed the algorithm, performed the result analysis and experiments, and wrote and revised the paper. All authors contributed to the article and approved the submitted version.
